# Perspectives of the General Public Regarding Government Policies in Combating the COVID-19 Pandemic

**DOI:** 10.7759/cureus.28332

**Published:** 2022-08-24

**Authors:** Sumeru Thapa Magar, Joseph Hankins, Saima Batool, Syeda Areeba Hussain Kazmi, Rana Inamullah Zafar, Mehjabeen Ahmad, Faraz Saleem, Izza Iftikhar, Muhammad Saqlain Qavi, Muhammad Abu Zar Ghaffari

**Affiliations:** 1 Cardiovascular, Janaki Medical College and Teaching Hospital, Tribhuvan University, Kathmandu, NPL; 2 Internal Medicine, Universidad Latina de Costa Rica, San Pedro, CRI; 3 Internal Medicine, Hameed Latif Hospital, Lahore, PAK; 4 Internal Medicine, Karachi Medical and Dental College, Karachi, PAK; 5 Internal Medicine, Army Medical College, Rawalpindi, Rawalpindi, PAK; 6 Internal Medicine, Nishtar Medical University, Multan, PAK; 7 Internal Medicine, Akhtar Saeed Medical and Dental College, Lahore, PAK; 8 Internal Medicine, Quaid-e-Azam Medical College, Lahore, PAK

**Keywords:** public response, public perception, health policy, pandemic, public health

## Abstract

Background

The world has been hit with one of the deadliest pandemics in history. This pandemic has affected almost all countries and more than 50 million people globally. This paper takes an in-depth look at all government policies that were developed to prevent the spread of coronavirus disease 2019 (COVID-19) in Pakistan and the perception of the general population regarding these policies. This study aims to provide help for policymakers to consider as they build more resilient regions.

Methodology

A descriptive, cross-sectional study was conducted online in Pakistan from April 2021 to September 2021. A self-administered questionnaire was distributed after obtaining informed consent. A sample size of 200 was calculated using the World Health Organization’s (WHO) sample size calculator. The data analysis was performed using SPSS version 23 (IBM Corp., Armonk, NY, USA), and the data are presented in the form of frequency tables, bar charts, and pie charts.

Results

Of the 200 respondents, 71% were satisfied with government policies, and 28.5% were not satisfied. A total of 66.5% of respondents thought that government policies were effective in combating COVID-19. More than half (80%) of the participants believed that government policies have reduced the rate of COVID-19 spread, while 20% thought that the policies did not help in reducing the rate of spread. Overall, 96% of the respondents supported quarantine as a good step taken by the Pakistan government to prevent COVID-19.

Conclusions

The satisfaction rate of the general public regarding government policies developed to combat COVID-19 was 71.5%. Government strategies should be improved to counter the impact of large-scale epidemics, and further studies are required to educate the public. With an already fragile healthcare system, this can have long-lasting issues in developing countries as the world might be expecting a new challenge in the form of *monkeypox*.

## Introduction

Coronavirus disease 2019 (COVID-19) has affected almost every dimension of human life, ranging from health to social, psychological, economic, and financial domains [1]. The world was soon disrupted by the emergence of COVID-19, with the hardest hit countries, such as America and Europe, reporting the highest death count of 178,378 deaths [2]. Because the virus was new and of unknown etiology, governments across the world enforced travel restrictions to and from China [3].

Governments across the world imposed a complete lockdown on their entire population (20% of the global population) with the closure of public places and transport, isolation of suspected cases, and establishment of quarantine centers [4]. In India, after the successful control of the first wave of COVID-19, the government granted relaxation in interventions. Due to this relaxation, along with negligent public behavior and the mutant strain (B.1.1.617) that emerged, the second wave created havoc with 0.2 million cases [5,6].

Government agencies were unable to understand the behavior of people during the pandemic. A national action plan for COVID-19 was devised by the Ministry of Health with the aim of effective preparedness and efficient response by ensuring all safeguards were in place to prevent an outbreak of COVID-19. The identification and activation of available financial and other resources to ensure maximum preparedness and response, provision of supplies, and infection prevention and control in healthcare settings were also a part of this plan [7]. New strategies were devised to implement health measures for travelers and strengthen readiness capacity to rapidly identify, diagnose, and treat cases, including identifying contacts with tracing and follow-up, as well as minimizing the community spread of the virus in Pakistan. Quarantine centers were established in the urban centers of Pakistan with the help of Pakistan’s armed forces, and a robust surveillance system was developed throughout Pakistan [3].

The lockdown became a great threat to the economic and financial stability of the country. Policy frameworks were drafted in an attempt to prevent, detect, and respond immediately to confirmed cases by federal and provincial governments [8]. A vast majority of the people believe that their government and the country’s citizens are not doing enough and underestimate the degree to which others in their country support strong behavioral and policy responses to the pandemic. The lack of satisfaction with government policies is associated with higher levels of worry among the public and the resultant fall in the public’s compliance rate [9].

The rationale of this survey is to focus on the current state of the COVID-19 pandemic in Pakistan by addressing the limitations and challenges in emergency preparedness and critically reviewing the results of the plan of action devised by the government of Pakistan. The willingness to cooperate and adopt health-protective behaviors during pandemics can only be truly predicted by measuring the public’s reaction to the crisis and coping strategies.

## Materials and methods

This study was conducted among the general population living in and around Lahore as an online survey. The study was conducted from April 2021 to September 2021. A descriptive, cross-sectional study design was used for this study. Informed consent was obtained from all study participants.

A sample size of 200 was calculated with the help of the World Health Organization’s (WHO) sample size calculator by maintaining a 95% confidence level and a 5% margin of error. A non-probabilistic convenience sampling technique was used for data collection. A self-administered questionnaire was designed using Google Forms. It was distributed through social media platforms and direct email solicitation. Questionnaires were distributed among national groups created on social media to spread COVID-19 information and concerns. Only people over the age of 18 and those who could read and understand English and could fill out the mediums online were included in the study sample. Those under the age of 18 and who were not willing to participate were excluded from the study.

We conducted a detailed literature review to draft a questionnaire. The draft questionnaire was pilot tested among 25 participants (the results of which were excluded from the study) and was sent to multiple senior researchers for evaluation, incorporating all appropriate recommendations into the questionnaire. Informed consent was obtained and kept confidential, and no personally identifiable information was collected or stored. The structured questionnaire was finalized and consisted of 33 questions.

The questionnaire mainly included the following aspects: (1) a brief description of the study and informed consent; (2) demographic variables such as age, gender, education level, area of residence, family income, and occupation; (3) perception and beliefs of the public regarding the COVID-19 pandemic; (4) perspectives of the government’s response to cope with the pandemic and testing strategy; (5) awareness and willingness to wear masks and social distancing; (6) perceptions of lockdown strategies; (7) closures of educational institutes and public centers; (8) sources of communication and media regarding COVID-19; (9) perception and beliefs of the public about the government’s vaccination program; (10) diagnostic and testing facilities; and (11) perceptions of the government’s strategy to meet healthcare needs by capacity enhancement of public hospitals and involvement of the private healthcare system.

The collected data were entered, coded, and analyzed using SPSS version 23 (IBM Corp., Armonk, NY, USA). The results were assessed using descriptive statistics, and means with their standard deviation (SD) were presented for continuous variables such as age. Categorical variables were reported as frequencies and percentages. Data are presented in the form of frequency tables, bar charts, and pie charts. Quantitative variables are presented as means and SDs.

## Results

This study included a total of 200 participants, of whom 46% (n = 92) were males and 54% (n = 108) were females. The mean age of the study population was 25.83 years. Approximately 91% (n = 183) of the study participants were aged between 18 and 30 years. The study population comprised predominantly people living in densely populated urban areas (51.5%; n = 103), and 48.5% were from large urban centers (n = 97). Over half of the participants were doctors by profession (n = 102), 5% were teachers, 4.5% were office workers, 3% were businesspersons, and 73 (36.5%) belonged to other professions. The majority of study participants were graduates (70%; n = 140). Overall, 40% of the respondents had a family income greater than PKR 100,000 per month. The demographics and characteristics of the participants and individual p-values are presented in Table 1.

**Table 1:** Socio-demographics and characteristics of the study sample. Number of participants: 200. SD: standard deviation; PKR: Pakistani rupee

Variables	Frequency (%)	
Mean age (years) ± SD	25.83 ± 6.82	
Gender		
Male	92 (46)	
Female	108 (54)	
Residence		
Big urban center	97 (48.5)	
Suburban	103 (51.5)	
Occupation		
Businesspersons	6 (3)	
Doctor	102 (51)	
Office worker	9 (4.5)	
Teacher	10 (5)	
Any other	73 (36.5)	
Education		
Matric	1 (0.5)	
Intermediate	38 (19)	
Graduate	140 (70)	
Masters and above	21 (10.5)	
Monthly income (PKR)		
>25,000	28 (14)	
25,001–50,000	33 (16.5)	
50,001–75,000	22 (11)	
75,000–100,000	36 (18)	
<100,000	81 (40.5)	

Respondents’ perception of COVID-19

**Figure 1:**
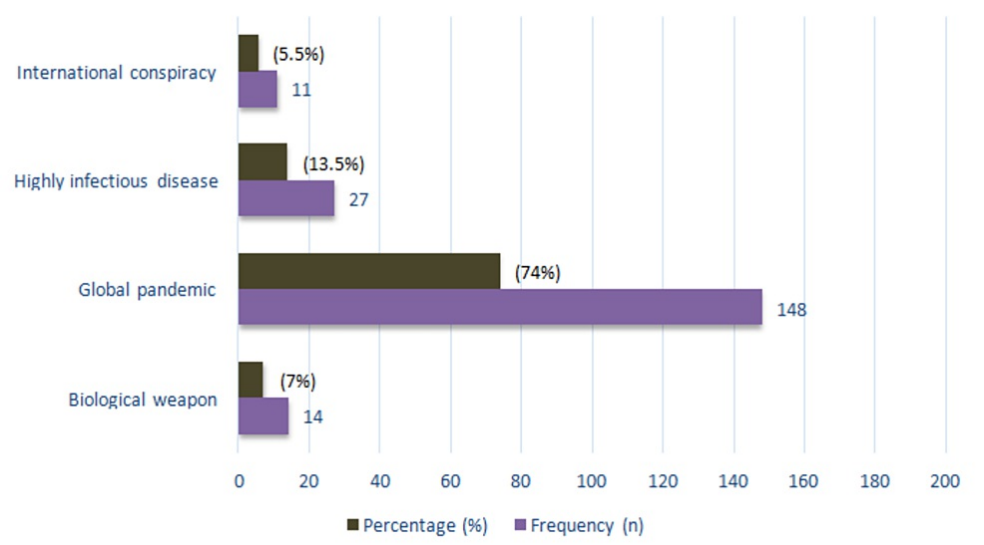
Respondents’ perception of the COVID-19 virus. COVID-19: coronavirus disease 2019

A large segment of our study population was found to be well aware of the existence of COVID-19 as a pandemic (74%; n = 148) and found it to be a major problem for the community (7%; n = 14) who perceived it as a biological weapon, whereas 5.5% considered it as an international conspiracy, and others professed it as a highly infectious disease (n = 27). The results are shown in Figure 1.

Perspectives of the public regarding government policies in combating COVID-19

Of the 200 respondents, 71.5% (n = 143) were satisfied with the government’s policies, and 28.5% (n = 57) were not satisfied. A total of 66.5% (n = 133) of participants agreed that government policies were effective, and 33.5% (n = 67) did not agree that the policies were effective in combating the virus. More than half (80%; n = 160) of the respondents thought that government policies had reduced the rate of COVID-19 spread, while 20% (n = 40) thought that the policies had not reduced the rate of spread.

Lockdown policies and social distancing

Only 16.5% (n = 33) of the respondents thought that the lockdown policies were not effective against COVID-19. More than half (65%; n = 130) of the respondents thought that smart lockdown was better than complete lockdown, while 35% favored complete lockdown. Of the 200 respondents, 97% (n = 195) agreed with the government’s decision to make wearing masks and social distancing mandatory, while only five (2.5%) respondents did not agree with the decision. Overall, 91% of the respondents believed that wearing masks helped them escape COVID-19, while 9% believed that wearing masks did not help them. A total of 79.5% (n = 159) of the respondents favored the government’s decision to impose restrictions on public transportation, while 20.5% (n = 41) opposed it. Moreover, 81% of the participants were satisfied with the traveling restrictions placed by the government to manage COVID-19, while 19% were not satisfied. More than half (n = 160) of the participants believed that government guidelines for educational institutions were effective, whereas 20% did not agree.

Of the 200 respondents, 62.5% (n = 125) agreed with the government’s decision to close educational institutions and instituting online classes, while 37.5% (n = 75) disagreed with this decision.

Approximately half (56%; n = 108) of the participants believed that the government’s decision to strictly impose standard operating procedures (SOPs) on the general public was effective. More than half (87%) of the respondents thought that the government was effectively providing health education messages to the public through the media, whereas 13% were not satisfied. Moreover, 96% of the respondents believed that quarantine was a good step taken by the government to prevent COVID-19.

Effectiveness of the vaccination program

Of the 200 participants, 97.5% agreed that the government’s vaccination program was a good step toward managing COVID-19. A total of 178 (89%) participants thought that the government should make vaccination mandatory for all, and 11% thought that it should not be mandatory for all. More than half of the participants (93.5%; n = 187) believed that the government should invest more in Pakistan regarding vaccines instead of importing them from abroad, while 6.5% (n = 13) thought that the government should buy foreign vaccines. Overall, 96% (n = 192) of the respondents agreed that everyone should be vaccinated free of cost.

Public’s perception regarding polymerase chain reaction testing

Of the 200 respondents, 95.5% (n = 191) agreed that it was a good step taken by the government to test the family members or contacts of a COVID-19-positive case for screening, while 4.5% (n = 9) disagreed. A total of 194 (97%) respondents thought that free polymerase chain reaction (PCR) testing by the government was a good decision. More than half (76%; n = 152) of the 200 respondents believed that the government’s diagnostic capacity and home testing services had improved, while 24% (n = 48) believed that the services needed to be improved. Overall, 63% (n = 126) of the respondents believed that the government had increased its healthcare capacity, while 37% (n = 74) were not satisfied. Moreover, 83 (41.5%) thought that the government hospitals were capable of carrying the COVID-19 load, while more than half (58.5%; n = 117) thought that the hospitals could not carry the load. Out of the 200 respondents, 95.5% (n = 191) agreed that the government should collaborate with the private sector, while 4.5% (n = 9) disagreed.

Table 2 describes all the questions asked from the participants regarding their perspectives on the government’s policies in combating the COVID-19 pandemic.

**Table 2:** Perspectives of the public regarding government’s policies in combating the COVID-19 pandemic. COVID-19: coronavirus disease 2019; SOP: standard operating procedure; PCR: polymerase chain reaction

Serial number	Questions	Frequency (%)		P-value
		Agree	Disagree	
1	Are you satisfied with government policies on COVID-19?	143 (71.5)	57 (28.5)	0.0003
2	Do you think government policies are effective?	133 (66.5)	67 (33.5)	0.0350
3	Do you think that government policies have reduced the rate of COVID-19?	160 (80)	40 (20)	0.0021
4	Do you think lockdown is an effective policy against COVID-19?	167 (83.5)	33 (16.5)	0.0445
5	Do you think smart lockdown is better than complete lockdown?	130 (77.8)	37 (22.2)	0.0089
6	Do you think government’s decision of wearing masks and keeping social distance is a good decision?	195 (97.5)	5 (2.5)	0.0001
7	Do you think wearing a mask has helped you escape COVID-19?	182 (91)	18 (9)	0.0070
8	The government has imposed restrictions on public transport as well. Do you think it is a good step?	159 (79.5)	41 (20.5)	0.0490
9	Do you think government’s guidelines issued for educational institutions were effective?	160 (80)	40 (20)	0.0079
10	Do you agree with the government’s decision to close educational institutions and online learning classes?	125 (62.5)	75 (37.5)	0.0134
11	Do you think SOPs set by the government for markets and public places are effective to combat the disease?	150 (75)	50 (25)	0.0712
12	Do you think restrictions imposed by government on restaurants are effective to combat the disease?	162 (81)	32 (19)	0.0063
13	Are you satisfied by the government’s air travel restrictions to contain COVID-19 virus?	162 (81)	38 (19)	0.0465
14	Do you think that the government is strictly enforcing SOPs to the general public by imposing fines and imprisonments?	108 (56)	92 (44)	0.0487
15	Do you think the government has effectively provided health education messages to the general public through TV and other communication media?	174 (87)	26 (13)	0.0001
16	Do you think government’s vaccination program is a good step?	195 (97.5)	5 (2.5)	0.0053
17	Do you think that government should make vaccination mandatory for every individual?	178 (89)	22 (11)	0.0065
18	Do you think government should not buy vaccination doses from other countries and invest more in vaccine preparation in Pakistan?	187 (93.5)	13 (6.5)	0.0009
19	Do you think the government should administer vaccine to everyone free of cost?	192 (96)	8(4)	0.0307
20	Do you think it is a good initiative by the government to test all contacts and family members of COVID-19-positive cases?	191 (95.5)	9 (4.5)	0.0892
21	Do you think the government should continue conducting PCR free of cost for everyone?	194 (97)	6 (3)	0.0088
22	Do you think the government has significantly increased the laboratory diagnostic capacity and has provided home testing facility for COVID-19?	152 (76)	48 (24)	0.0294
23	Do you think quarantine is a good decision by the government?	192 (96)	8 (4)	0.0156
24	Do you think the government has significantly increased healthcare system capacity according to the needs of the nation?	126 (63)	74 (37)	0.0737
25	Do you think that government hospitals have sufficient healthcare staff and facilities to treat COVID-19 patient load?	83 (41.5)	117 (58.5)	0.0090
26	Do you think the government should collaborate with the private sector to provide better health facilities to its people?	191 (95.5)	9 (4.5)	0.0002

## Discussion

COVID-19 is a serious public health emergency with severe adverse implications for the population, healthcare systems, and economies globally [10]. Centralized and professional leadership, democratic and accountable political culture, liberated civil society, and broad social participation are the key features of disease control in a country [11].

The results highlight the broader impact of the COVID-19 pandemic on the public’s overall health and well-being, outside personal infection, and their satisfaction regarding governmental policies in combating the coronavirus infection [12].

This study was conducted to determine people’s perspectives regarding government policies to combat the COVID-19 pandemic. In this study, out of the 200 respondents, 14 (7%) thought of it as a global pandemic, 148 (74%) thought of it as a biological weapon, 27 (13.5%) thought of it as a highly infectious disease, and 11 (5.5%) thought of it as an international conspiracy. In Bangladesh, out of 240 respondents, 290 (85%) thought COVID-19 was a global pandemic [13].

In our study, around one-third of the participants were not satisfied with government policies to combat COVID-19, whereas a large majority were satisfied. In another survey, out of 200 people, 110 (55%) responded that the government was not reacting sufficiently, 50 (25%) did not trust their government policies, and 40 (20%) were satisfied with government policies [14]. In the current study, 160 (80%) thought that the government policies had reduced the rate of coronavirus infection, and 40 (20%) thought that government policies were not effective in reducing the rate of COVID-19 infection. In another survey in the UK, the data were collected from several interventions in 41 countries, out of which 77% of the respondents agreed with the reduction of COVID-19 infection and decreased mortality rate due to policies developed by their government [15].

Out of 167 respondents who favored lockdown, 130 (77.9%) thought that smart lockdown was better than complete lockdown, and 37 (22.1%) thought that complete lockdown was better. In another study in Italy, out of 100,000 respondents, 30,000 (30%) thought that the complete lockdown was a better option, while 70,000 (70%) considered smart lockdown to be a better option [16]. In this study, out of the 200 respondents, 195 (97.5%) agreed with the government’s decision to wear masks and social distancing mandatory, while five (2.5%) disagreed with the decision.

In this survey, out of the 200 respondents, 182 (91%) believed that wearing masks helped them escape COVID-19; however, 18 (9%) believed that wearing masks did not help them. In Singapore, 32.5% of doctors, 48.7% of nurses, and 77% of administrative personnel thought that a simple mouth-to-nose protection mask would be sufficient to prevent infection [17]. Out of the 200 respondents, 159 (79.5%) favored the government’s decision to impose restrictions on public transportation, while 41 (20.5%) opposed it. However, in another survey in Poland, 90% resigned or limited their usage of public transportation based on precautions imposed by the government [18].

Out of the 200 respondents, 185 (92.5%) agreed with government policies to put restrictions on public places, markets, and social gatherings to prevent COVID-19, while 15 (7.5%) did not agree. In the United States, 74-83% of the decline in foot traffic was due to the formal restriction of social gatherings [19]. Therefore, communities should continue to discourage large gatherings, identify and test social contacts, and isolate confirmed cases to help control the COVID-19 pandemic.

In this study, out of the 200 respondents, 125 (62.5%) agreed with the government’s decision to close educational institutions and online classes, and 75 (37.5%) disagreed with the decision. However, in another study in Indonesia, out of 66 participants, 62 (93.9%) students were involved in online classes, and only four (6.1%) had problems joining the online sessions [20]. Out of the 200 respondents, 150 (75%) believed that SOPs set by the government for markets and public places were effective in combating the disease, and 50 (25%) believed that SOPs were not effective.

Out of the 200 participants, 108 (56%) thought that the government strictly imposed SOPs on the public, while 92 (44%) did not agree. In another study in the UK, out of 300 respondents, 210 (70%) thought that the government was strictly imposing SOPs, while 90 (30%) did not agree [21]. Out of the 200 people, 195 (97.5%) agreed that vaccination programs were a good step, and five (2.5%) did not agree, while in Australia, out of 1,420, 1,143 (80%) agreed that getting themselves vaccinated for COVID-19 would be a good way to protect themselves against COVID-19, while 277 (20%) were unaware [22].

In this survey, out of the 200 people, 178 (89%) said that vaccination should be mandatory, while 22 (11%) did not like the idea. In another survey held in Australia, out of 1,420 respondents, 355 (25%) self-reported chronic diseased people, and 820 (58%) private health insurance agreed to have mandatory vaccinations for everyone [22]. In this survey, 187 (93.5%) people thought the government should invest more in Pakistan regarding vaccination, and 13 (6.5%) said the government should buy foreign vaccines. Out of the 200 respondents, 192 (96%) agreed that the government should vaccinate everyone free of cost, while eight (4%) disagreed. In another survey in India, out of 1,000 respondents, 840 (84%) reacted in a positive tone, and 160 (16%) answered in a negative tone [23].

In this study, 191 (95.5%) said that it is a good step to test the family members of the patients with a positive COVID-19 test, while nine (4.5%) did not agree. Out of the 200 respondents, 194 (97%) said that performing PCR tests free of cost was a good step, while six (3%) did not consider it good. In another survey in Oxford (UK), 96% considered the free PCR test to be a good initiative. In this study, out of the 200 respondents, 152 (76%) agreed that the government had improved the diagnostic capacity and home testing services, while 48 (24%) disagreed. In another study in Bangladesh, out of 1,066 respondents, 875 (82%) strongly agreed, 123 (12.4%) agreed, 40 (3.1%) neither agreed nor disagreed, eight (0.9%) disagreed, and 20 (1.6%) strongly disagreed [24].

Out of the 200 respondents, 192 (96%) considered quarantine a good step for the effective prevention of COVID-19 infection, while eight (4%) disagreed. In Jordan, of 5,057 participants, 4,800 (95%) perceived the benefits of home quarantine [25]. In this survey of 200 people, 126 (63%) agreed that the government had increased healthcare capacity, while 74 (37%) disagreed. In Bangladesh, out of 1,066 respondents, 800 (75.4%) agreed, while 266 (24.6%) disagreed that their government had increased healthcare capacity [24]. During the current COVID-19 pandemic, hospitals have reported a breakdown of services when the surge of patients in need of treatment and ventilator support outpaced available capacities. Out of the 200 respondents, 83 (41.5%) agreed that government hospitals have sufficient healthcare services to handle the COVID-19 load, while 117 (58.5%) disagreed. In the UK, 75% agreed to have sufficient healthcare facilities [26].

Out of the 200 people, 191 (95.5%) agreed that the government should collaborate with the private sector, while nine (4.5%) disagreed, while in another survey in Norway, citizens’ satisfaction with democracy and their collaboration with the private sector increased from 57% to 72%, which was a very high rating internationally [27]. In this survey, out of the 200 respondents, 162 (81%) were satisfied with the travel restrictions by the government for better handling of COVID-19, while 38 (19%) were not satisfied. In another survey in Paris, out of 1,010 respondents, 470 (46.6%) completely agreed, 240 (23.6%) partially agreed, 130 (12.2%) neither agreed nor disagreed, 80 (8%) partially disagreed, and 80 (7.5%) completely disagreed with the travel restrictions imposed by the government to halt the spread of infection [28].

Limitations

Like every study, our study also had some limitations. First, due to a strict lockdown, the study questionnaire was distributed via online platforms, including social media. Internet penetration varies with age groups, income level, and educational status. Also, as students of medicine, we had a higher reach to the healthcare community on our social media platforms compared to non-healthcare professionals. Therefore, this could be a challenge in depicting the results, especially when the perception of government policies could also vary widely among these different groups, as mentioned in Table 1. However, the latter point can also provide a better opportunity in understanding the concerns of the public across the occupational strata and especially involving the segment of the society which was affected way more than the rest of the professionals. Second, our sample size of 200 also presents a challenge to adequately predict the public perception at large and difficult to generalize the results to the whole country.

However, we consider that our study has been successful in addressing the specific concerns that the policymakers would face in the future when developing long-term strategies, keeping in mind the perspective and, more importantly, the acceptance of the public. This study also provides a road map, in light of which more studies can be conducted with larger sample sizes and different segments of society.

## Conclusions

The current global economic and health situation is a point of concern for the general population with fragile health systems and low-quality healthcare services. A vast majority of the public was satisfied with the government policies developed to cope with the COVID-19 pandemic. However, a large proportion was not satisfied with the management of the resources, including materials, equipment, information, and human resource. Moreover, a large section of the public expressed dissatisfaction with the effectiveness of the government’s policies, particularly the closure of educational institutes and public places.

This reinforces the need to take measures and address pandemic preparedness by developing more strategic policies to cater to the resurge of such catastrophes in the future. These comprehensive findings can help in developing an emergency and a long-term plan at the national level on pandemic education, managing resources, ensuring hospital preparedness, addressing the rumors and conspiracy theories, and improving the overall healthcare system that can counter the impact of large-scale epidemics. With the ever-changing environment we are living in, COVID-19 has precluded yet more challenges, the latest in the form of *monkeypox*, more strategic policies need to be included in the future. Therefore, this study takes a more significant spot in shaping public health policies as long-term objectives can only be achieved with better public participation. Further studies in Pakistan can yield a holistic conclusion on the overall knowledge levels and perceptions of the different segments of the society toward government policies.

## Appendices

**Table 3:** Study questionnaire. COVID-19: coronavirus disease 2019; SOP: standard operating procedure; PCR: polymerase chain reaction

Section I: Sociodemographic profile						
Serial number	Questions	Options				
1	Gender:	Male	Female			
2	Age (years):	18–30	30–40	40–50	50–60	60 or above
3	Area of residence:	Lahore	Outside Lahore			
4	Occupation:	Doctor	Teacher	Office worker	Businessman	Any other
5	Education:	Matric	Intermediate	Graduate	Master or above	
6	Family income in Pakistani Rupees/Month:	<25,000	25,001–50,000	50,001–75,000	75,001–100,000	100,001 or above
Section II: People’s perspective regarding government policies to combat COVID-19						
Serial number	Questions	Options				
7	What do you think about the COVID-19 virus ?	Hoax	International conspiracy	Global pandemic	Biological weapon	Highly infectious disease
8	Are you satisfied with government’s policy on COVID-19?	Yes	No			
9	Do you think the government’s policies are effective?	Yes	No			
10	Do you think that government’s policies have reduced the rate of COVID-19?	Yes	No			
11	Do you think lockdown is an effective policy against COVID-19?	Yes	No			
12	Do you think smart lockdown is better than complete lockdown?	Yes	No			
13	Do you think government’s decision of wearing mask and keeping social distance is a good decision?	Yes	No			
14	Do you think wearing a mask has helped you escape COVID-19?	Yes	No			
15	The government has imposed restrictions on public transport as well. Do you think it is a good step?	Yes	No			
16	Do you think government’s guidelines issued for educational institutions were effective?	Yes	No			
17	Do you agree with the government’s decision to close educational institutions and online learning classes?	Yes	No			
18	Do you think SOPs set by the government for markets and public places are effective to combat the disease?	Yes	No			
19	Do you think restrictions imposed by government on restaurants are effective to combat the disease?	Yes	No			
20	Are you satisfied by the government’s air travel restrictions to contain the COVID-19 virus?	Yes	No			
21	Do you think that government is strictly enforcing SOPs to the general public by imposing fines and imprisonments?	Yes	No			
22	Do you think the government has effectively provided health education messages to the general public through TV and other communication media?	Yes	No			
23	Do you think government’s vaccination program is a good step?	Yes	No			
24	Do you think that the government should make vaccination mandatory for every individual?	Yes	No			
25	Do you think the government shouldn’t buy vaccination doses from other countries and invest more in vaccine preparation in Pakistan?	Yes	No			
26	Do you think the government should administer vaccine to everyone free of cost?	Yes	No			
27	Do you think it is a good initiative by the government to test all contacts and family members of COVID-19-positive cases	Yes	No			
28	Do you think the government should continue conducting PCR free of cost for everyone?	Yes	No			
29	Do you think the government has significantly increased the laboratory diagnostic capacity and has provided home testing facility for COVID-19?	yes	no			
30	Do you think quarantine is a good decision of the government?	Yes	No			
31	Do you think the government has significantly increased healthcare system capacity according to the needs of the nation?	Yes	No			
32	Do you think that government hospitals have sufficient healthcare staff and facilities to treat the COVID-19 patient load?	Yes	No			
33	Do you think the government should collaborate with the private sector to provide better health facilities to its people?	Yes	No			

## References

[1] Raza SH, Haq W, Sajjad M (2020). COVID-19: a psychosocial perspective. Front Psychol.

[2] Noreen N, Siddiqui SW, Niazi SU (2020). COVID-19 outbreak in Pakistan; a situational analysis. J Emerg Dis Virol.

[3] Javed B, Sarwer A, Soto EB, Mashwani ZU (2020). Is Pakistan's response to coronavirus (SARS-CoV-2) adequate to prevent an outbreak?. Front Med (Lausanne).

[4] Afzal MS, Khan A, Qureshi UU (2021). Community-based assessment of knowledge, attitude, practices and risk factors regarding COVID-19 among Pakistanis residents during a recent outbreak: a cross-sectional survey. J Community Health.

[5] Bhuyan A (2021). Experts criticise India's complacency over COVID-19. Lancet.

[6] (2021). WHO: coronavirus disease dashboard of India. https://COVID19.who.int/region/searo/country/in.

[7] Ladiwala ZF, Dhillon RA, Zahid I, Irfan O, Khan MS, Awan S, Khan JA (2021). Knowledge, attitude and perception of Pakistanis towards COVID-19; a large cross-sectional survey. BMC Public Health.

[8] Abid K, Bari YA, Younas M, Tahir Javaid S, Imran A (2020). Progress of COVID-19 epidemic in Pakistan. Asia Pac J Public Health.

[9] Dryhurst S, Schneider CR, Kerr J (2020). Risk perceptions of COVID-19 around the world. J Risk Res.

[10] Li B, Yang J, Zhao F (2020). Prevalence and impact of cardiovascular metabolic diseases on COVID-19 in China. Clin Res Cardiol.

[11] Yeh MJ, Cheng Y (2020). Policies tackling the COVID-19 pandemic: a sociopolitical perspective from Taiwan. Health Secur.

[12] Meena S (2020). Impact of novel Coronavirus (COVID-19) pandemic on travel pattern: a case study of India. Indian J Sci Technol.

[13] Islam SM, Bodrud-Doza M, Khan RM, Haque MA, Mamun MA (2020). Exploring COVID-19 stress and its factors in Bangladesh: a perception-based study. Heliyon.

[14] Fetzer T, Witte M, Hensel L (2020). Global behaviors, perceptions, and the emergence of social norms at the onset of the COVID-19 pandemic. PsyArXiv.

[15] Brauner JM, Mindermann S, Sharma M (2021). Inferring the effectiveness of government interventions against COVID-19. Science.

[16] Di Porto E, Naticchioni P, Scrutinio V (2020). Partial lockdown and the spread of Covid-19: lessons from the Italian case. IZA Discussion Paper.

[17] Driessche KV, Mahlobo PZ, Venter R (2021). Face masks in the post-COVID-19 era: a silver lining for the damaged tuberculosis public health response?. Lancet Respir Med.

[18] Przybylowski A, Stelmak S, Suchanek M (2021). Mobility behaviour in view of the impact of the COVID-19 pandemic—public transport users in Gdansk case study. Sustainability.

[19] Cronin CJ, Evans WN (2020). Private precaution and public restrictions: what drives social distancing and industry foot traffic in the COVID-19 era?. NBER Working Papers.

[20] Agung AS, Surtikanti MW, Quinones CA (2020). Students’ perception of online learning during COVID-19 pandemic: a case study on the English students of STKIP Pamane Talino. Soshum.

[21] Dhami MK, Weiss-Cohen L, Ayton P (2020). Are people experiencing the 'pains of imprisonment' during the COVID-19 lockdown?. Front Psychol.

[22] Seale H, Heywood AE, Leask J, Sheel M, Durrheim DN, Bolsewicz K, Kaur R (2021). Examining Australian public perceptions and behaviors towards a future COVID-19 vaccine. BMC Infect Dis.

[23] Praveen SV, Ittamalla R, Deepak G (2021). Analyzing the attitude of Indian citizens towards COVID-19 vaccine - a text analytics study. Diabetes Metab Syndr.

[24] Bodrud-Doza M, Shammi M, Bahlman L, Islam AR, Rahman MM (2020). Psychosocial and socio-economic crisis in Bangladesh due to COVID-19 pandemic: a perception-based assessment. Front Public Health.

[25] Al-Sabbagh MQ, Al-Ani A, Mafrachi B (2022). Predictors of adherence with home quarantine during COVID-19 crisis: the case of health belief model. Psychol Health Med.

[26] Donker T, Bürkin FM, Wolkewitz M (2021). Navigating hospitals safely through the COVID-19 epidemic tide: predicting case load for adjusting bed capacity. Infect Control Hosp Epidemiol.

[27] Christensen T, Lægreid P (2020). Balancing governance capacity and legitimacy: how the Norwegian government handled the COVID-19 crisis as a high performer. Public Adm Rev.

[28] Kallbekken S, Sælen H (2021). Public support for air travel restrictions to address COVID-19 or climate change. Transp Res D Transp Environ.

